# Enhanced efficacy of gemcitabine in combination with anti-CD20 monoclonal antibody against CD20+ non-Hodgkin's lymphoma cell lines *in vitro *and in *scid *mice

**DOI:** 10.1186/1471-2407-5-103

**Published:** 2005-08-18

**Authors:** Mitchell R Smith, Indira Joshi, Fang Jin, Coleman Obasaju

**Affiliations:** 1 Department of Medical Oncology Fox Chase Cancer Center 333 Cottman Avenue Philadelphia, PA19111, USA; 2 Lilly Research Laboratories, Indianapolis, IN, USA

## Abstract

**Background:**

Despite exciting new targeted therapeutics against non-Hodgkin's lymphoma (NHL), chemotherapy remains a cornerstone of therapy. While purine nucleoside analogs have significant activity in low grade NHL, the pyrimidine nucleoside analog gemcitabine has been less extensively studied, but has important activity. Use of the anti-CD20 monoclonal antibody rituximab in combination with chemotherapy for B-NHL is becoming prevalent in clinical practice, but has not been extensively studied in pre-clinical models.

**Methods:**

We have tested the activity of gemcitabine ± rituximab *in vitro *and in *scid*/human NHL xenograft models. We used two t(14;18)+, CD20+ follicular B cell NHL cell lines, DoHH2 a transformed NHL line and WSU-FSCCL isolated from pleural fluid of a patient with indolent NHL.

**Results:**

Gemcitabine is cytotoxic to DoHH2 and WSU-FSCCL cells *in vitro*, and the IC_50 _is 2–3 fold lower in the presence of rituximab. Apoptosis is also enhanced in the presence of rituximab. Clearance of NHL cells from ascites in *scid *mice is prolonged by the combination, as compared with either agent alone. Most importantly, survival of *scid *mice bearing human NHL cells is significantly prolonged by the combination of gemcitabine + rituximab.

**Conclusion:**

Based on our pre-clinical data showing prolonged survival of mice bearing human lymphoma cell line xenografts after treatment with gemcitabine + anti-CD20 antibody, this combination, expected to have non-overlapping toxicity profiles, should be explored in clinical trials.

## Background

Non-Hodgkin's lymphoma (NHL) is increasing in incidence and is now the fifth most common malignancy in the U.S. Despite novel targeted biologic treatment options, chemotherapy remains an important component of therapy. Furthermore, most patients with indolent lymphoma and at least half of all patients with aggressive NHL are not cured [1]. Improved therapeutic approaches are needed.

Gemcitabine is a pyrimidine nucleoside analog with clinical anti-cancer activity. Purine nucleoside analogs such as fludarabine, cladribine and pentostatin have been extensively studied and have significant activity against certain non-Hodgkin's lymphoma subtypes, particularly indolent forms. Though less well studied, an increasing body of data indicates activity of gemcitabine against lymphoma, both Hodgkin's and NHL [2-7]. The precise place of gemcitabine in the therapeutic armamentarium for NHL remains to be elucidated.

The chimeric anti-CD20 monoclonal antibody rituximab is active as a single agent in B cell NHL [8]. In addition, it may sensitize cells to the action of chemotherapeutic and other biologic agents pre-clinically [9-12], as well as in patients [13,14]. While mechanisms of rituximab action include direct apoptotic induction, complement activation and antibody dependent cytotoxicity, which of these is important may depend on the experimental conditions, and the relative importance in patients remains to be determined (reviewed in [15]). The same mechanisms, as well as intracellular signaling [16], may account for chemosensitization, but again the exact means by which this occurs in patients remains to be fully elucidated. One previous report has demonstrated *in vitro *sensitization of aggressive B cell NHL cell lines to gemcitabine by rituximab [10]. Here we extend these *in vitro *results to additional human CD20+ lymphoma cell lines that carry the t(14:18) translocation, perhaps more analogous to the clinical use of rituximab. More importantly, we demonstrate that gemcitabine + rituximab enhances survival *in vivo *in a human B-NHL cell line/*scid *mouse xenograft model.

## Methods

### Cell culture and growth assay

Cells are incubated under standard conditions and cell numbers are determined by methyl thiazol tetrazoliumbromide (MTT) assay as before [17]. Briefly, DoHH2 [18], a t(14;18)+ transformed lymphoma cell line, was obtained from Deutsche Sammlung von Mikroorganismen und Zellkulturen GmbH (DMSZ, German Collection of Microorganisms and Cell Cultures, Braunschweig, Germany). WSU-FSCCL, as previously reported [19], was isolated from pleural fluid of a patient with follicular grade I lymphoma, contains t(14;18), is EBV negative and behaves in *scid *mice as a more indolent disease [17], although it does contain a c-myc translocation.

### Anti-CD20 monoclonal antibody

Chimeric anti-CD20 (clone C2B8, rituximab) obtained from IDEC (San Diego, CA), and gemcitabine (generously supplied by Lilly, Indianapolis, IN) were injected intraperitoneally (ip).

### Apoptosis and cell cycle

Cell cycle was analyzed by DNA content per cell, by propidium iodide (PI) staining of nuclei from hypotonically lysed cells [20]. Apoptosis was determined by dual staining of 1 × 10^5 ^intact cells in 100 μl calcium binding buffer containing 5 μl of fluoroscein isothiocyanate (FITC)-labeled annexin V (Pharmingen) and 5 μg/ml PI for 15 minutes in the dark, followed by analysis by flow cytometry (FACScan).

### Poly(ADP-ribose) polymerase (PARP) cleavage assay

DoHH2 and WSU-FSCCL cells were lysed in cold radioimmunoprecipitation assay (RIPA) buffer [21] containing 100 μg phenylmethanesulfonyl fluoride (PMSF)/ml and 1 μg aprotinin/ml for 30 min on ice, pelleted and the supernatant separated by 4–20% SDS-PAGE. Transfer was to Immobilon-P, blocked with 1% casein-0.04% Tween-20 and probed with anti-PARP C2–10 antibody (Trevigen, Gaithersburg, MD). Secondary antibody was horseradish peroxidase labeled anti-mouse IgG (1:3000) for 30 min, detected by chemiluminescence and Hyperfilm ECL (Amersham, Piscataway, NJ). Densitometry utilized NIH Image 1.61 software.

### *Scid*/human xenograft

Female CB17 *scid *mice were bred, housed and treated in the Fox Chase Cancer Center Laboratory Animal Facility under an approved protocol. Mice 4–8 weeks old were injected ip with either 1 × 10^7 ^WSU-FSCCL cells or 5 × 10^6 ^DoHH2 cells. Mice were observed daily and euthanized when they appeared ill. Lymphoma involving diffuse adenopathy, splenomegaly, infiltration of liver and bone marrow, along with ascites developed in untreated mice with each model, at 8–11 weeks with WSU-FSCCL and 4–6 weeks with DoHH2. Cells were collected sequentially from ascites by peritoneal washings and analyzed, or mice were followed for survival. For ascites clearance, mice bearing DoHH2 received 20 μg rituximab intraperitoneally (ip) on day 3 after lymphoma cell injection and/or gemcitabine 120 μg/gm ip on day 4, while mice bearing WSU-FSCCL received 100 μg rituximab intraperitoneally (ip) on day 7 after lymphoma cell injection, and/or gemcitabine 120 μg/gm ip on day 8. For survival, mice bearing DoHH2 received 5 μg rituximab on days 2, 9 and 16 and/or gemcitabine 120 μg/gm ip on days 3, 10 and 17.

## Results

### Cell growth inhibition by gemcitabine and rituximab

**Figure 1:**
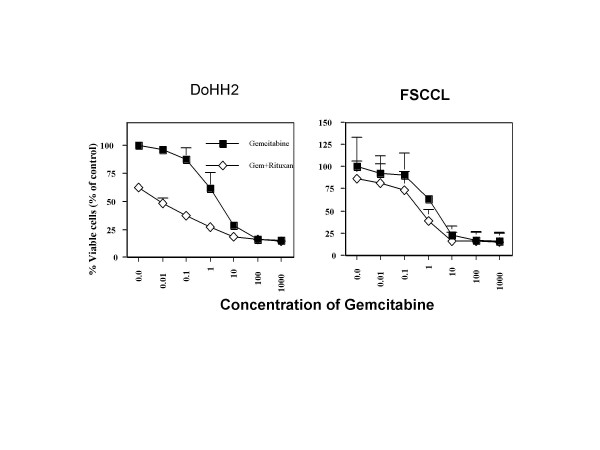
Cytotoxicity of rituximab and gemcitabine. WSU-FSCCL or DoHH2 cells incubated with varying concentrations of gemcitabine, and 4 hr later ± 20 μg/ml rituximab. Cytotoxicity is expressed as percentage of untreated control cells at 72 hr.

Both DoHH2 and WSU-FSCCL cells are growth inhibited by gemcitabine with an IC_50 _of 1 nM after 72 hr incubation (Figure 1). Addition of rituximab alone or in combination with gemcitabine had little effect on WSU-FSCCL cells *in vitro*. In contrast, DoHH2 cells are growth inhibited about 35% by 20 μg/ml rituximab. The maximal rituximab effect is seen at 1 μg/ml. This is accord with data that CD20 sites are saturated at this level, while effective serum levels in patients are felt to be 25 μg/ml. Thus, there is essentially no dose response at clinically achievable levels. This precludes the calculation of synergy by the standard approach [22], as has been concluded by other investigators as well [23]. Because of our prior experience that oligonucleotides can interfere with drug and antibody uptake, we separated addition of the two agents by 4 hours to preclude direct physical interaction of the antibody and drug, however, no effect of order of addition within 4 hours was observed with rituximab and gemcitabine (data not shown).

### Cell cycle alteration by gemcitabine and rituximab

**Figure 2:**
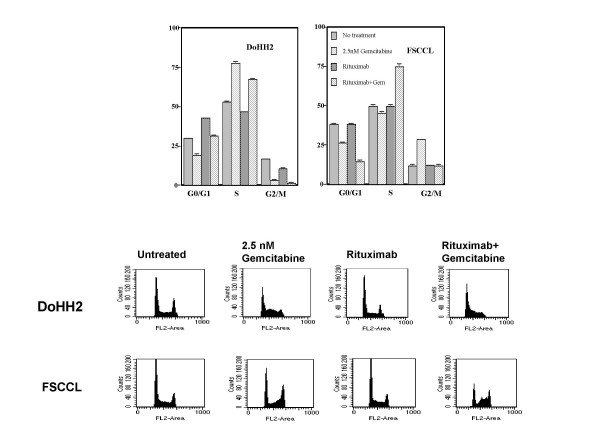
Effect of gemcitabine and rituximab on cell cycle. Cells (0.25 × 10^6^/ml) incubated ± 20 μg/ml rituximab for 4 hr, then ± 2.5 nM gemcitabine for 24 hr. Cells stained with propidium iodide, analyzed by flow and cell cycle parameters calculated (ModFit LT program). A typical flow result is also shown (Figure 2A).

Continuous exposure to 2.5 nM gemcitabine led to accumulation of DoHH2 cells in the S phase, as has been generally reported for gemcitabine [24]. WSU-FSCCL cells, however, accumulated in the G2/M phase of the cell cycle (Figure 2). Although rituximab alone had no significant cell cycle effects on either cell line, nor on the S phase accumulation of DoHH2 cells, the combination of gemcitabine + rituximab led to S phase accumulation of WSU-FSCCL cells. Statistical analysis of S phase block in WSU-FSCCL cells comparing 2.5 nM gemcitabine alone versus gemcitabine plus rituximab revealed p < 0.001. Thus, the combination had different cell cycle effects on WSU-FSCCL than did either agent alone. The precise basis of this change is unclear, though presumably involves an alteration in the S phase DNA damage sensor, which appears to involve the ATM gene [25].

### Apoptosis induced by gemcitabine and rituximab

**Figure 3:**
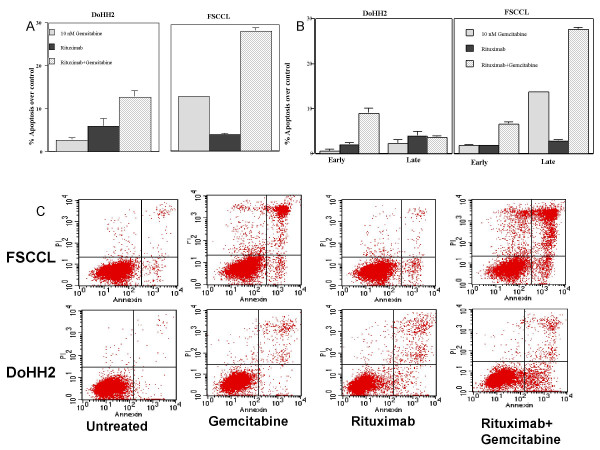
Apoptosis induced by gemcitabine and rituximab. Cells treated as in figure 2, except with 10 nM gemcitabine, then stained with annexin V and propidium iodide. Top left panel (A) is total annexin V positive cells. Top right  panel (B) shows early apoptosis (annexin+/PI-, left) and late apoptosis (annexin+/PI+, right). A typical dot plot (C) is also shown.

Apoptosis was assayed by annexin V staining, which detects the altered location of phosphatidyl serine to the outer surface of the cell membrane. Apoptosis was induced at modest levels by gemcitabine or rituximab, but significantly more apoptosis was induced by the combination in both cell lines studied (Figure 3). When apoptosis was separated into early apoptosis, in which cells still exclude propidium iodide (PI), and late apoptosis where cells are PI permeable, early apoptosis is induced by the combination in DoHH2 cells, whereas both early and late apoptosis are demonstrated in WSU-FSCCL cells.

**Figure 4:**
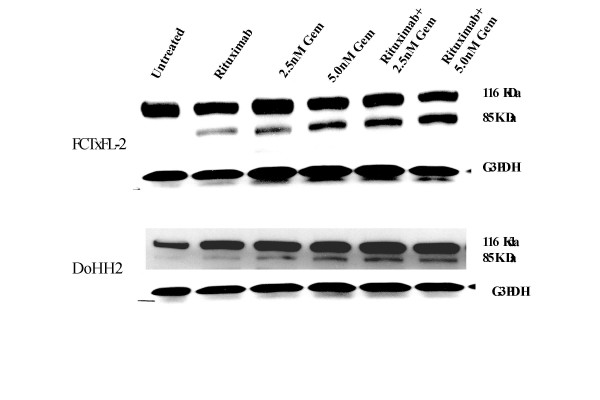
PARP cleavage induced by gemcitabine and rituximab. Cells treated as in figure 2, but for 48 hr with either 2.5 or 5 nM gemcitabine, the cell lysates analyzed by Western using anti-PARP (C2–10, Trevigen, Inc), which detects both intact PARP (116 kD) and the apoptotic marker PARP cleavage fragment (85 kD), or G3PDH as a loading control.

Apoptosis was also assessed by cleavage of PARP, which occurs when the apoptotic pathway is activated, eventually leading to cleavage of caspase 3 and of other downstream proteins including PARP. In each cell line, rituximab and gemcitabine result in PARP cleavage, with additional cleavage using the combination (Fig 4).

### *In vivo *efficacy of gemcitabine ± rituximab

**Figure 5:**
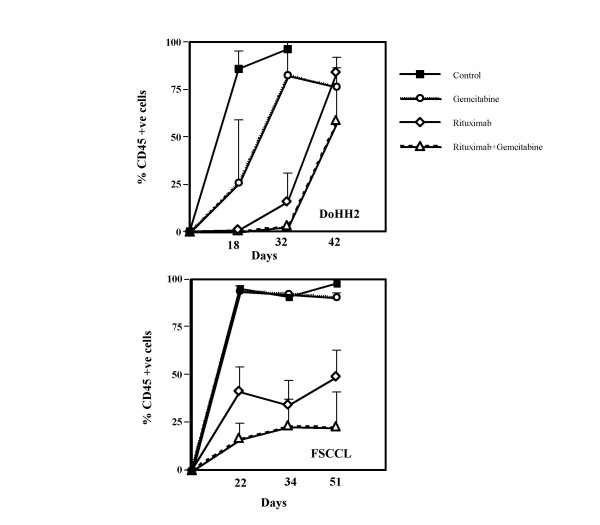
Clearance of NHL cells from ascites by treatment with gemcitabine and rituximab. *Scid *mice were injected intraperitoneally (ip) with 5 × 10^6 ^DoHH2 cells (A, top) or 10 × 10^6 ^WSU-FSCCL cells (B, bottom). Mice received 20 μg rituximab (ip) on day 3 and 120 μg gemcitabine/gm body weight (ip) on day 4 after injection of DoHH2 cells. Ascites was collected on day 18, 32 and 42. Untreated *scid/*DoHH2 mice died prior to day 42. Because of the slower development of lymphoma with WSU-FSCCL, mice were treated with 100 μg rituximab (ip) on day 7 and 120 μg gemcitabine/gm body weight (ip) on day 8 after injection of WSU-FSCCL cells. Ascites was collected on days 22, 34 and 51. Cells from ascites were stained with PE anti-human CD45 antibody and analyzed by flow cytometry.

We initially screen for efficacy of therapy in our model by assessing the prevention of growth of cells in ascites fluid [11,17]. The lymphomas grow as bulky mesenteric nodes with development of hepatosplenomegaly, as well as diffuse adenopathy elsewhere, but not with measurable disease. While ascites represents only a small part of the animals' disease burden, it can be repeatedly sampled as an indicator of overall tumor in a mouse. Given the differing rates of growth of the cell lines in mice, treatment was started on day 3 after DoHH2 cell injection and day 7 after WSU-FSCCL cell injection. For DoHH2 (Figure 5A), gemcitabine delayed growth, so that at day 18 there were fewer lymphoma cells in the ascites fluid, however, by day 32 there was no longer a difference. Rituximab is effective in this model, although cells do eventually reaccumulate in ascites. There is a trend to longer time to recurrence of lymphoma cells with combined therapy. In the WSU-FSCCL model (Figure 5B), while gemcitabine alone had no effect, it did enhance the rituximab-mediated delay in lymphoma cell re-growth.

**Figure 6:**
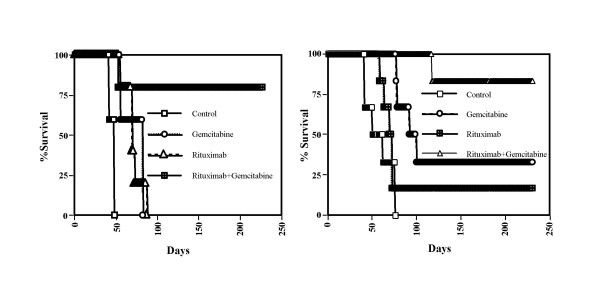
Survival of *scid/*DoHH2 mice after treatment with gemcitabine and rituximab. Two experiments in which *scid *mice injected with DoHH2 cells were treated ± 5 μg rituximab on days 2, 9 and 16 and ± gemcitabine (120 μg/gm) on days 3, 10 and 17. Mice were followed for survival and euthanized when ill. For comparison of rituximab + gemcitabine to rituximab alone, p = 0.04 (left) and p = 0.01 (right). Overall, the combination cured 9/11 mice, compared with 2/11 for gemcitabine and 1/11 with rituximab.

The most important endpoint for treatment efficacy, since there is not an externally measurable lesion, is survival of mice bearing the human lymphoma cell lines. We have performed duplicate experiments using mice injected with DoHH2 cells (Figure 6). At the gemcitabine dose of 120 μg/gm (~ 2.4 mg/mouse), used in previous reports [26] weekly for 3 doses, there was modest prolongation of median survival, with 0/5 and 2/6 long-term survivors in the two experiments. We used sub-maximally tolerated doses of rituximab, 5 μg per mouse, which had modest therapeutic effect (1/11 long-term survivors combined). Combination therapy with these two agents at the same dose and schedule, however, markedly prolonged survival (p = 0.04, left; p = 0.01, right for rituximab + gemcitabine vs rituximab), and cured 9 of 11 mice. The surviving mice were euthanized at the end of the experiment and found to be histologically negative for lymphoma.

## Discussion

This report demonstrates the anti-lymphoma activity of the pyrimidine analog gemcitabine *in vitro *and in a *scid *mouse human lymphoma xenograft model. Rituximab is an active therapy for CD20+ NHL as a single agent and in combination with some biologic and chemotherapeutic agents [9-12]. The generalizability and mechanism of chemosensitization by rituximab has not been fully explored. Evidence for several mechanisms of rituximab mediated lymphoma cell death has been presented including direct induction of apoptosis, complement mediated killing and antibody-dependent cell mediated cytotoxicity (ADCC). Much of the data addressing these processes comes from *in vitro *systems, and the relevance to activity in patients remains uncertain. Our *in vitro *data in this report rely only on direct apoptosis, as we do not add human serum as a complement source nor effector cells. Direct apoptosis can be affected by degree of crosslinking of the antibody, which in turn can be controlled by humoral or cell surface molecules. Complement activity may be altered by inhibitory factors and may differ between human serum in vitro and mouse complement in murine models. We have found that crosslinking does enhance the degree of apoptosis after rituximab treatment in our two cell lines, while addition of complement has little effect (data not shown). ADCC depends on effector cells, and even the precise effector cells remain uncertain. *Scid *mice have residual granulocytes and NK cells, and depletion of these cells can abolish rituximab efficacy [27]. To understand the mechanisms of resistance to rituximab will require more complete knowledge of which of these mechanisms of action is or are most important in patients [15]. Recent data suggests that rituximab can alter intracellular signaling, even without inducing apoptosis, in ways that can sensitize cells to chemotherapy effects [16].

Our results demonstrate that the combination of gemcitabine and rituximab inhibits NHL cell growth, induces apoptosis in these cells, and, most importantly, is effective in prolonging survival of mice bearing human t(14;18)+ lymphoma cells. Prior reports have shown additive effects of gemcitabine and rituximab in aggressive NHL cell lines *in vitro *[10]. In that report, aggressive NHL cell lines that were relatively resistant to both gemcitabine and rituximab were pre-treated with rituximab for 24 hours and then treated with gemcitabine for 18 hours and found to have modest increases in hypodiploid cells and PI positive cells after PI staining.

The cell cycle changes seen after gemcitabine treatment alone, and in combination with rituximab, are cell line dependent. WSU-FSCCL cells, in contrast to most cells, are not blocked in S phase by gemcitabine alone, but are after rituximab is added. This may reflect altered intracellular signaling that could restore a putative S phase DNA damage sensor, such as the ATM pathway [25]. Similarly, induction of early versus late apoptosis is also cell line dependent. Further exploration of the biochemical basis of these changes is warranted to better understand which of the many potential targets of a pyrimidine nucleoside analog are the important determinants of gemcitabine activity against NHL cells, and how these targets are affected by rituximab.

Concurrent treatment of B cell lymphoproliferative disorders (NHL and CLL) with rituximab and chemotherapy is becoming more common, and data suggests benefit in time to disease progression in indolent disease [14] and also in overall survival in aggressive disease [13]. Questions remain regarding the optimal way to combine rituximab and chemotherapy and whether therapeutic efficacy of specific chemotherapeutic agents is enhanced by rituximab. Pre-clinical studies of the interaction of chemotherapy agents and rituximab may provide insight to guide the development of appropriate clinical trials.

## Competing interests

There were no competing interests at the time this work was carried out. Subsequently, Dr. Obasaju has become employed by Lilly, maker of gemcitabine, but Lilly provided no support for this work.

## Authors' contributions

MS conceived the study, provided oversight of the lab and wrote the manuscript. IJ designed and carried out the experiments and critiqued the manuscript. FJ assisted in carrying out the experiments and critiqued the manuscript. CO helped in the conception of the studies and critiqued the manuscript.

## Pre-publication history

The pre-publication history for this paper can be accessed here:
